# MISIPI study: Melanoma ImmunoScore evaluation in patients treated with IPIlimumab

**DOI:** 10.1186/1479-5876-12-S1-P11

**Published:** 2014-05-06

**Authors:** Carlo Bifulco, Marilena Capone, Zip Feng, Gabriele Madonna, Ester Simeone, Marcello Curvietto, Nicola Mozzillo, Gennaro Ciliberto, Gerardo Botti, Bernard A Fox, Paolo A Ascierto

**Affiliations:** 1 Earle A. Chiles Research Institute – Providence Cancer Center, Portland, OR, USA; 2 Istituto Nazionale Tumori Fondazione “G. Pascale”, Napoli, Italy

## Background

Increasing evidence has supported the hypothesis that cancer development is influenced by the host immune system. Immune infiltrates of the primary tumors and metastases are independent prognostic biomarkers and may represent predictive factors, suggesting that pre-therapeutic immune response determines the efficacy of different immunotherapies [1]. An immune-classification of tumors based on a scoring system of density and location of immune-cells within the tumor has been recently proposed in human colorectal cancers, where a high density of CD3+, CD8+ memory T lymphocytes in the primary tumor is associated with long disease free and overall survival and low risk of relapse and metastasis [2,3]. The impact of the immune score needs to be evaluated in other tumor types given the universal importance of the immune system in cancers. We have focused on the prognostic and predictive value of the immunoscore in patients with advanced melanoma treated with Ipilimumab in the previous 2-3 years, and correlated it with outcomes and response to the treatment.

## Materials and methods

We have collected 190 FFPE metastatic samples from melanoma patients treated with Ipilimumab, using the last lesion excised before the treatment: lymph node involvement or distant metastatic sites. We are characterizing the immunescore with the expression of CD3, CD8, CD20, Foxp3, by immunohistochemistry and evaluated correlation with clinical outcome. Serial sections (3-4 micron in thickness) have been stained with each marker and counterstained with H/E. Moreover, one section has been stained with a multiplex of all markers including tumor marker for melanoma (S100). All slides will be digitized on a Leica SCN-400 system, and the results will be quantified by assessing the density of different cellular immune population at the center and invasive margin of the tumor using a digital images analysis application (Definiens Architect XD). The markers expression will be subsequently matched with the most important clinical information about all the patients.

## Results

We have considered the clinical benefit from treatment with Ipilimumab as the parameter for evaluating the effectiveness of immunescore. The treatment has been considered as effective if a patient met a partial or complete response, or had stable disease for more than 6 months from the start of therapy, or with an overall survival > 1 year. The evaluation of prognostic and predictive power of the immunoscore, in patients with metastatic melanoma, treated with ipilimumab, are nearing completion and definition.

## Conclusion

We believe that the immunescore is key as prognostic and predictive markers for immunotherapies in metastatic melanoma.
